# Trapping of yFACT at 3’ ends of genes is not a universal characteristic of yeast versions of Bryant-Li-Bhoj syndrome histone H3 mutants

**DOI:** 10.17912/micropub.biology.001384

**Published:** 2024-10-25

**Authors:** Joseph S. Beard, Lillian K. Francis, Reece C. Forrest, Agustin Kalinowski, Jackson C. Parks, William H. Griffin, Caroline L. Tackett, Andrea A. Duina

**Affiliations:** 1 Biology Department, Hendrix College

## Abstract

Bryant-Li-Bhoj syndrome (BLBS) is associated with germline mutations in the genes encoding human histone H3.3. While to date 70 H3.3 mutants have been associated with BLBS, the molecular mechanisms underpinning this condition remain undefined. We recently showed that in yeast the H3-L61R BLBS mutant causes trapping of yFACT at 3’ ends of genes, raising the possibility that this defect could be a contributing factor to disease across all H3-BLBS mutants. Here, we show that of nine additional yeast H3-BLBS mutants analyzed, only one causes yFACT 3’ end-trapping, thus indicating that this defect is not a universal feature of H3-BLBS mutants. We also present additional phenotypic data that could provide insights into the molecular mechanisms contributing to BLBS in human patients.

**Figure 1. Effects of yeast histone H3-BLBS mutants on Spt16-gene interactions and on phenotypes associated with chromatin-related processes:**
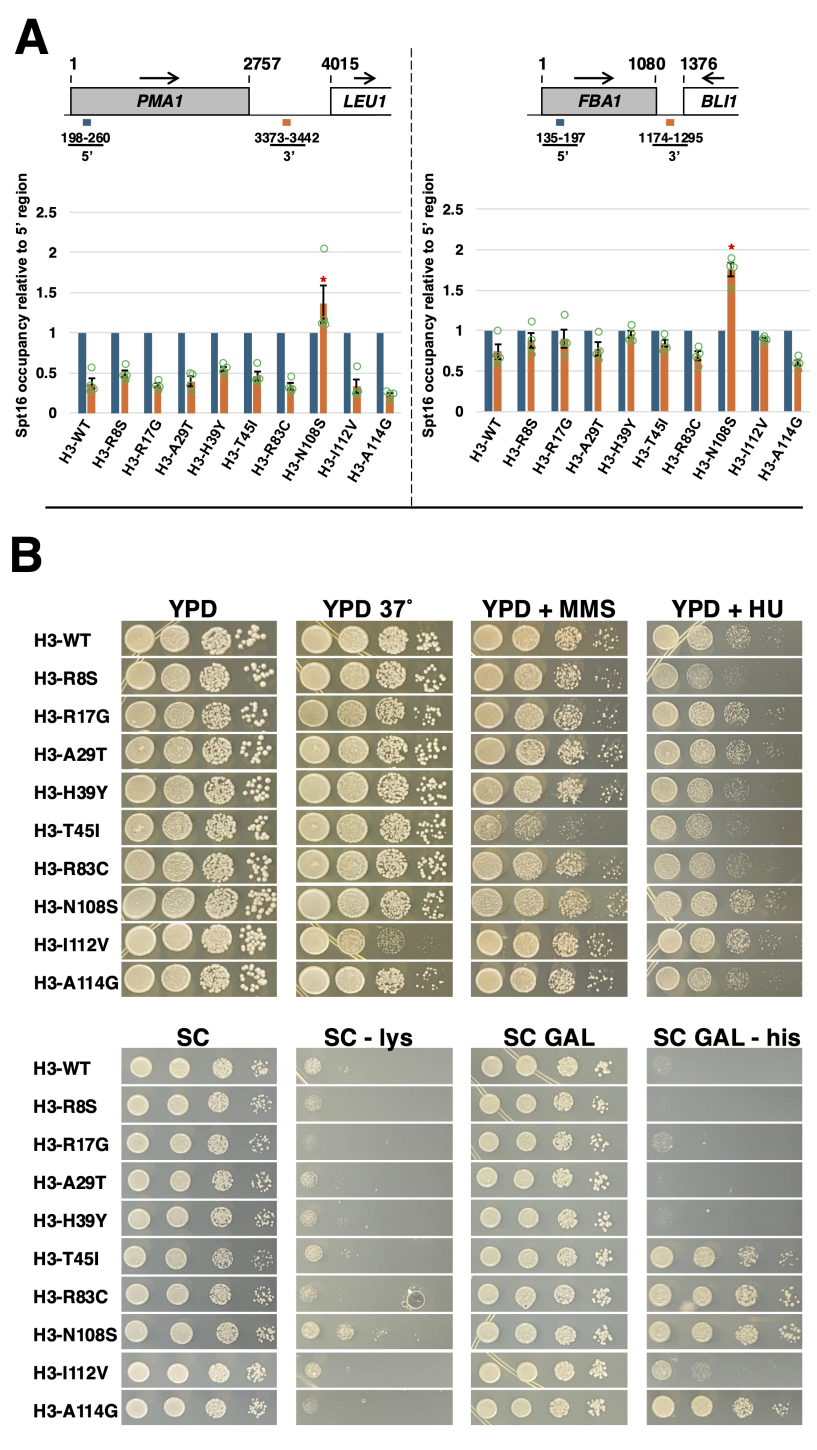
**(A)** ChIP assays to assess Spt16 occupancy across the *PMA1* and *FBA1* genes in strains expressing various histone H3-BLBS mutants. On the top diagram, the “1” indicates the start of the coding region of each of the two genes tested and the blue and orange bars underneath correspond to the 5’ and 3’ regions amplified by qPCR, respectively. Arrows show the direction of transcription. For each strain, bar graphs show Spt16 occupancy levels relative to occupancy at the 5’ region, which is arbitrarily set to 1. In each case, data are presented as mean ± S.E.M. from four independent experiments, with individual data points shown by green-bordered circles. Statistically significant differences relative to H3-WT cells are indicated by red asterisks (Student’s *t* -tests, *P* < 0.05). **(B)** Growth tests were performed by spotting 10X-dilution series of each strain on the indicated growth media. Plates were incubated at 30˚C (except for the YPD 37˚ plate) for two days for the YPD, YPD 37˚, YPD + MMS and SC plates, and three days for the YPD + HU, SC – lys, SC GAL, and SC GAL – his plates. Growth patterns were confirmed in an independent experiment. The strains used for the experiments shown in both panels are yADP89 and yADP156-yADP164.

## Description

Bryant-Li-Bhoj syndrome (BLBS) is a recently described human neurodevelopmental and neurodegenerative condition caused by the presence of mutant versions of the histone H3.3 protein (one of the three canonical histone H3 isoforms expressed in human cells along with histones H3.1 and H3.2) (Bryant et al. 2024; Layo-Carris et al. 2024). To date, 70 distinct variants of H3.3 have been associated with BLBS and the correlated amino acid substitutions are found across the entire length of the protein (Maver et al. 2019; Bryant et al. 2020; Okur et al. 2021; Layo-Carris et al. 2024). In human cells, H3.3 is expressed from two differentially regulated genes, *H3F3A* and *H3F3B* , and mutations in both genes have been identified in BLBS patients. Given the extensive number, nature, and widespread locations of BLBS amino acid substitutions across H3.3, it is likely that several processes are impacted by the collection of these mutants, and deciphering which ones are causative for the condition represents a significant challenge. Progress on this front has come from a recent study using a human-derived model system showing increased expression and chromatin deposition of the *H3.3B* p.L48R BLBS mutant and concurrent changes in expression of genes with roles in normal neuronal function (Sangree *e* t al. 2024).

To contribute to the understanding of the basic molecular processes affected by H3-BLBS mutants, we recently analyzed the BLBS amino acid substitution L61R using the budding yeast *Saccharomyces cerevisiae* as the model system. Yeast cells express a single version of canonical histone H3 that is 90% identical to human H3.3 (McBurney et al. 2016) and, like H3.3, it is expressed from two distinct genes, *HHT1* and *HHT2* . In our studies, we found that whereas the H3-L61R mutant causes lethality when expressed as the sole source of histone H3 in yeast, it allows viability when co-expressed with wild-type histone H3 but confers several phenotypes indicative of transcription and chromatin defects (Johnson et al. 2015; Pablo-Kaiser et al. 2022). The most striking defect relates to the trapping of the histone chaperone complex yFACT (yeast Facilitates Chromatin Transcription/Transactions, a heterodimer composed of Spt16 and Pob3 (recently reviewed in (Formosa and Winston 2020; Zhou et al. 2020; Jeronimo and Robert 2022)) at the 3’ end of transcribed genes, a defect we have attributed to an impairment in yFACT dissociation from genes following transcription (Pablo-Kaiser et al. 2022). Since in human patients as well as in metazoan model systems mutations or conditions that impair function of Spt16 homologs have been associated with neurodevelopmental defects (Bina et al. 2020; Ma et al. 2023; Wang et al. 2023), it is possible that the *H3F3A* p.L61R BLBS mutant causes disease by trapping the FACT complex at 3’ ends of genes, thereby reducing the availability of FACT for critical chromosomal processes.

To assess if yFACT 3’ end-trapping could be a universal defect shared across all yeast H3-BLBS mutants, we generated yeast strains expressing nine additional histone H3-BLBS mutants from their endogenous *HHT2* locus and tested them for defects in yFACT-gene interactions using the chromatin immunoprecipitation (ChIP) assay followed by qPCR. Since these strains also harbor a deletion of the *HHT1* gene, these experiments assessed the effects of the H3-BLBS mutants when present as sole source of histone H3 in cells. The nine mutants were selected among the thirty-seven mutants that were reported in the first extensive investigation related to H3.3 mutants and neurodevelopmental disorders (Bryant et al. 2020) based on the criteria that each was found in more than one patient (with the additional patient(s) having either the same or different amino acid substitution compared to the one we tested) and that in each case the wild-type residue is conserved between the yeast and human histone H3 proteins. (We note that one of the mutants that fulfills this criteria, H3-A114G, was not included in later publications related to BLBS (Bryant et al. 2024; Layo-Carris et al. 2024) as subsequent analyses raised the possibility that it might have been identified as a result of a sequencing artifact, but since this has not been fully established (E. Bhoj, personal communication) we included it in this study).

As shown in panel A of the figure, of the nine mutants we tested, only H3-N108S shows significant trapping of the Spt16 subunit of yFACT at the 3’ end of the highly and constitutively expressed *PMA1* and *FBA1* genes. Whereas these experiments do not allow us to directly compare the degree of the trapping defect between H3-N108S and H3-L61R since the studies on the latter mutant were performed in cells that also express wild-type histone H3, the H3-L61R mutant is likely to be an overall stronger mutant since the degree of its effects are comparable to those seen in H3-N108S cells despite the fact that the H3-L61R cells also express wild-type histone H3 (Pablo-Kaiser *et al.* 2022). Interestingly, in the tertiary structure of the nucleosome, N108 is located in the vicinity of residues that comprise the ISGI (Influences Spt16-Gene Interactions) region of the nucleosome, which L61 is also a part of and whose integrity is required for proper yFACT dissociation from 3’ ends of genes (Nguyen *et al.* 2013; Nyamugenda *et al.* 2018). Whereas residues H3-T45, -I112, and -A114 are also relatively near the ISGI region, the BLBS substitutions at these locations do not appear to affect yFACT-gene interactions, possibly because they do not alter the structure and/or function of this region in ways that cause yFACT 3’-end trapping. The fact that eight of the nine H3 mutants we tested do not cause trapping of yFACT at the 3’ ends of *PMA1* and *FBA1* indicates that abnormal yFACT-gene interactions is not a defect conferred by all H3-BLBS mutants.

To gain additional insights into the functional features of H3-BLBS mutants, we conducted growth tests to assay for defects in other chromosomal-based processes. As shown in the upper portion of panel B in the figure, the H3-R8S, -H39Y, -T45I, -R83C, and -A114G mutants show mild sensitivity to the DNA-replication inhibitor hydroxyurea (HU), suggesting that these histone mutants may interfere with DNA replication processes. H3-T45I also confers sensitivity to the DNA-damaging agent methyl methanesulfonate (MMS), indicating that this mutant may interfere with DNA repair processes as well. In addition, one mutant, H3-I112V, confers sensitivity to the high temperature of 37˚C, possibly indicating that this amino acid substitution causes a decrease in histone H3 stability.

To assay for transcription and chromatin defects, we utilized two reporter systems. First, we assayed for the ability of the H3 mutants to suppress a defect in expression of the *LYS2* gene caused by the presence of a Ty *δ* element within the gene’s promoter region – suppression of this allele (known as *lys2-128* δ *)* is often caused by mutations that alter chromatin structure or otherwise affect the transcription machinery, allowing cells to grow on media lacking lysine and giving rise to a phenotype known as Spt ^-^ (Winston 1992) . Second, we monitored for cryptic intragenic transcription initiation events using the *GAL1p-FLO8-HIS3* reporter system (Cheung et al. 2008). In this system, the promoter of the *FLO8* gene has been replaced with the *GAL1* promoter and the coding region of the *HIS3* gene has been fused in frame with the *FLO8* open reading frame downstream from a cryptic TATA box – mutations that allow growth on media containing galactose and lacking histidine (His ^+^ phenotype) are indicative of chromatin defects permissive to intragenic transcription, such as those that interfere with transcription-coupled nucleosome reassembly. Whereas only the H3-N108S mutant causes a mild Spt ^-^ phenotype, four mutants – H3-T45I, -R83C, -N108S, and -A114G – cause strong His ^+^ phenotypes, suggesting that these H3-BLBS mutants may interfere with proper nucleosome reassembly or other chromatin processes. We note that in previous work we showed that the H3-L61R mutant causes both Spt ^-^ and His ^+^ phenotypes when expressed in conjunction with wild-type histone H3, thus indicating that this H3-BLBS mutant may also cause defects in chromatin and nucleosome reassembly processes (Pablo-Kaiser et al. 2022) .

Collectively, the results shown in panel A of the figure, combined with our previous studies (Pablo-Kaiser et al. 2022), demonstrate that while trapping of yFACT at 3’ ends of genes is a defect seen in the context of some yeast histone H3-BLBS mutants (i.e., H3-L61R and H3-N108S), it is not a universal feature of this class of histone H3 mutants. Extrapolating these findings to BLBS in human patients, our studies may point to FACT 3’-trapping as a possible contributing molecular mechanism driving the development of BLBS in the context of some, but not all, of the H3.3 BLBS mutants. The phenotypic analysis of the yeast histone H3-BLBS mutants shown in panel B, combined with a similar analysis we previously carried out for the H3-L61R mutant (Pablo-Kaiser et al. 2022), may also provide insights into the molecular mechanisms contributing to BLBS in human patients.

## Methods

***Yeast strains, genetic methods, and media:*** All yeast strains used in this study are *GAL2 ^+^* derivatives of the S288C strain background (Winston et al.1995). The strains expressing the histone H3-BLBS mutants were generated using the strategy we have previously described (Johnson et al. 2015; Duina and Turkal 2017). The genotypes of the resulting strains and the oligonucleotides used to generate each mutant are provided in the Reagents section. Standard genetic techniques and media preparation protocols have been described previously (Rose et al. 1990).

***Chromatin Immunoprecipitation (ChIP)/qPCR assays:*** ChIP/qPCR assays to assess occupancy of Spt16 across the *PMA1* and *FBA1* genes were carried out as previously described (Myers et al. 2011). The following primer sets were used for the qPCR analysis: *5’PMA1* , OAD394 and OAD395; *3’PMA1* , OAD383 and OAD384; *5’FBA1,* OAD419 and OAD420; *3’FBA1* , OAD423 and OAD424. Primer sequences have been provided in previous reports (Myers et al. 2011; Nguyen et al. 2013).

***Growth tests:*** Patches derived from single colonies of each of the strains tested were grown on YPD plates at 30˚C overnight and transferred to sterile deionized water and adjusted to a concentration of 1.37X10 ^7^ cells/ml. Ten-fold dilution series were then set up in microtiter plates, spotted on the plates shown in the figure (4μl for YPD-based plates and 6μl for SC-based plates), and incubated under the conditions indicated in the figure legend. Therefore, the most concentrated spots on YPD-based plates contained ~55,000 cells and those on SC plates contained ~82,500 cells. YPD + HU plates contained 150mM hydroxyurea and YPD + MMS plates contained 0.035% methyl methanesulfonate.

## Reagents

***Antibodies and drugs used in growth tests:*** ChIPs were carried out using polyclonal antibodies specific for yeast Spt16 . HU was obtained from Fisher Scientific, Cat. No. AC151681000, and MMS from Sigma Aldrich, Cat. No. 129925-5G.

***Saccharomyces cerevisiae strains used in this study:***

**Table:** 

**Strain Name**	Genotype	Source
yADP89	*MAT* **a** ; his3∆200;leu2∆;ura3*;lys2-128δ;KanMX4-GAL1pr-FLO8-HIS3;(hht1-hhf1)∆::LEU2;HHT2	(Johnson *et al.* 2015)
yADP156	*MAT* **a** ; his3∆200;leu2∆;ura3*;lys2-128δ;KanMX4-GAL1pr-FLO8-HIS3;(hht1-hhf1)∆::LEU2;hht2(H3-R8S)	This study
yADP157	*MAT* **a** ; his3∆200;leu2∆;ura3*;lys2-128δ;KanMX4-GAL1pr-FLO8-HIS3;(hht1-hhf1)∆::LEU2;hht2(H3-R17G)	This study
yADP158	*MAT* **a** ; his3∆200;leu2∆;ura3*;lys2-128δ;KanMX4-GAL1pr-FLO8-HIS3;(hht1-hhf1)∆::LEU2;hht2(H3-A29T)	This study
yADP159	*MAT* **a** ; his3∆200;leu2∆;ura3*;lys2-128δ;KanMX4-GAL1pr-FLO8-HIS3;(hht1-hhf1)∆::LEU2;hht2(H3-H39Y)	This study
yADP160	*MAT* **a** ; his3∆200;leu2∆;ura3*;lys2-128δ;KanMX4-GAL1pr-FLO8-HIS3;(hht1-hhf1)∆::LEU2;hht2(H3-T45I)	This study
yADP161	*MAT* **a** ; his3∆200;leu2∆;ura3*;lys2-128δ;KanMX4-GAL1pr-FLO8-HIS3;(hht1-hhf1)∆::LEU2;hht2(H3-R83C)	This study
yADP162	*MAT* **a** ; his3∆200;leu2∆;ura3*;lys2-128δ;KanMX4-GAL1pr-FLO8-HIS3;(hht1-hhf1)∆::LEU2;hht2(H3-N108S)	This study
yADP163	*MAT* **a** ; his3∆200;leu2∆;ura3*;lys2-128δ;KanMX4-GAL1pr-FLO8-HIS3;(hht1-hhf1)∆::LEU2;hht2(H3-I112V)	This study
yADP164	*MAT* **a** ; his3∆200;leu2∆;ura3*;lys2-128δ;KanMX4-GAL1pr-FLO8-HIS3;(hht1-hhf1)∆::LEU2;hht2(H3-A114G)	This study

*This allele is either *ura3-52* or *ura3∆0*

***Oligonucleotides used to generate histone H3 mutant strains:***

**Table:** 

**Encoded** **H3 mutant**	Name and Sequence (5’ to 3’)*	
H3-R8S	OAD881 (Forward): AACTAAACAAACAGCTagtAAATCCACTGGTGGTAA OAD882 (Reverse): TTACCACCAGTGGATTTactAGCTGTTTGTTTAGTT	
H3-R17G	OAD893 (Forward): ACTGGTGGTAAAGCCCCAgggAAACAATTAGCCTCC OAD894 (Reverse): GGAGGCTAATTGTTTcccTGGGGCTTTACCACCAGT	
H3-A29T	OAD885 (Forward):AGGCTGCCAGAAAATCCaccCCATCTACCGGTGGTG OAD886 (Reverse): CACCACCGGTAGATGGggtGGATTTTCTGGCAGCCT	
H3-H39Y	OAD887 (Forward): GTGGTGTTAAGAAGCCTtacAGATATAAGCCAGGTA OAD888 (Reverse): TACCTGGCTTATATCTgtaAGGCTTCTTAACACCAC	
H3-T45I	OAD851 (Forward): ACAGATATAAGCCAGGTAtTGTTGCCTTGAGAGAAA OAD852 (Reverse): TTTCTCTCAAGGCAACAaTACCTGGCTTATATCTGT	
H3-R83C	OAD895 (Forward): ATTTCAAGACCGACTTGtgtTTTCAATCTTCTGCTA OAD896 (Reverse): TAGCAGAAGATTGAAAacaCAAGTCGGTCTTGAAAT	
H3-N108S	OAD891 (Forward): TTTGTTTGAAGACACTagtCTGGCTGCTATTCACGC OAD892 (Reverse): GCGTGAATAGCAGCCAGactAGTGTCTTCAAACAAA	
H3-I112V	OAD889 (Forward): ACACTAATCTGGCTGCTgtgCACGCTAAGCGTGTTA OAD890 (Reverse): TAACACGCTTAGCGTGcacAGCAGCCAGATTAGTGT	
H3-A114G	OAD883 (Forward): ATCTGGCTGCTATTCACggtAAGCGTGTTACTATCC OAD884 (Reverse): GGATAGTAACACGCTTaccGTGAATAGCAGCCAGAT	

*Lower case letters represent nucleotides that differ from the corresponding wild-type sequence
